# A Pragmatic Method to Integrate Data From Preexisting Cohort Studies Using the Clinical Data Interchange Standards Consortium (CDISC) Study Data Tabulation Model: Case Study

**DOI:** 10.2196/46725

**Published:** 2023-12-21

**Authors:** Keiichi Matsuzaki, Megumi Kitayama, Keiichi Yamamoto, Rei Aida, Takumi Imai, Mami Ishida, Ritsuko Katafuchi, Tetsuya Kawamura, Takashi Yokoo, Ichiei Narita, Yusuke Suzuki

**Affiliations:** 1 Department of Public Health, School of Medicine, Kitasato University, Sagamihara, Japan; 2 Clinical Study Support Center, Wakayama Medical University Hospital, Wakayama, Japan; 3 Translational Research Institute for Medical Innovation, Osaka Dental University, Osaka, Japan; 4 Department of Medical Statistics, Osaka Metropolitan University, Osaka, Japan; 5 Clinical & Translational Research Center, Kobe University Hospital, Kobe, Japan; 6 Department of Medical Informatics and Clinical Epidemiology, Kyoto Prefectural University of Medicine, Kyoto, Japan; 7 Kidney Unit, National Hospital Organization Fukuokahigashi Medical Center, Fukuoka, Japan; 8 Kidney Unit, Medical Corporation Houshikai Kano Hospital, Fukuoka, Japan; 9 Division of Kidney and Hypertension, Department of Internal Medicine, Jikei University School of Medicine, Tokyo, Japan; 10 Division of Clinical Nephrology and Rheumatology, Graduate School of Medical and Dental Sciences, Niigata University, Niigata, Japan; 11 Department of Nephrology, Faculty of Medicine, Juntendo University, Tokyo, Japan

**Keywords:** data warehousing, data management, database integration, integrate multiple data sets, Study Data Tabulation Model, SDTM, Clinical Data Interchange Standards Consortium, CDISC

## Abstract

**Background:**

In recent years, many researchers have focused on the use of legacy data, such as pooled analyses that collect and reanalyze data from multiple studies. However, the methodology for the integration of preexisting databases whose data were collected for different purposes has not been established. Previously, we developed a tool to efficiently generate Study Data Tabulation Model (SDTM) data from hypothetical clinical trial data using the Clinical Data Interchange Standards Consortium (CDISC) SDTM.

**Objective:**

This study aimed to design a practical model for integrating preexisting databases using the CDISC SDTM.

**Methods:**

Data integration was performed in three phases: (1) the confirmation of the variables, (2) SDTM mapping, and (3) the generation of the SDTM data. In phase 1, the definitions of the variables in detail were confirmed, and the data sets were converted to a vertical structure. In phase 2, the items derived from the SDTM format were set as mapping items. Three types of metadata (domain name, variable name, and test code), based on the CDISC SDTM, were embedded in the Research Electronic Data Capture (REDCap) field annotation. In phase 3, the data dictionary, including the SDTM metadata, was outputted in the Operational Data Model (ODM) format. Finally, the mapped SDTM data were generated using REDCap2SDTM version 2.

**Results:**

SDTM data were generated as a comma-separated values file for each of the 7 domains defined in the metadata. A total of 17 items were commonly mapped to 3 databases. Because the SDTM data were set in each database correctly, we were able to integrate 3 independently preexisting databases into 1 database in the CDISC SDTM format.

**Conclusions:**

Our project suggests that the CDISC SDTM is useful for integrating multiple preexisting databases.

## Introduction

To use medical databases efficiently in clinical research, methods that efficiently integrate multiple databases must be established. The International Committee of Medical Journal Editors (ICMJE) requires researchers to include a data sharing statement when submitting a manuscript [1]. Moreover, there is a growing focus on the sharing of clinical research data and its uses. However, the current ICMJE statement makes no mention of specific data standards for data sharing. Therefore, a discussion regarding specific ways to share data collected in clinical research is needed.

Recently, several medical societies and research groups have formed registries and conducted large cohort studies. The integration of databases with the same disease focus enables the analysis of data for many end points and patients. The reanalysis of data comprising large cohorts such as pooled analysis has statistical power and derives more reliable results [2]. For example, the Premenopausal Breast Cancer Collaboration, supported by the National Cancer Institute in the United States, published the results of several studies that used pooled analysis methods to integrate data from 20 independent cohort studies [3].

**Figure 1.**
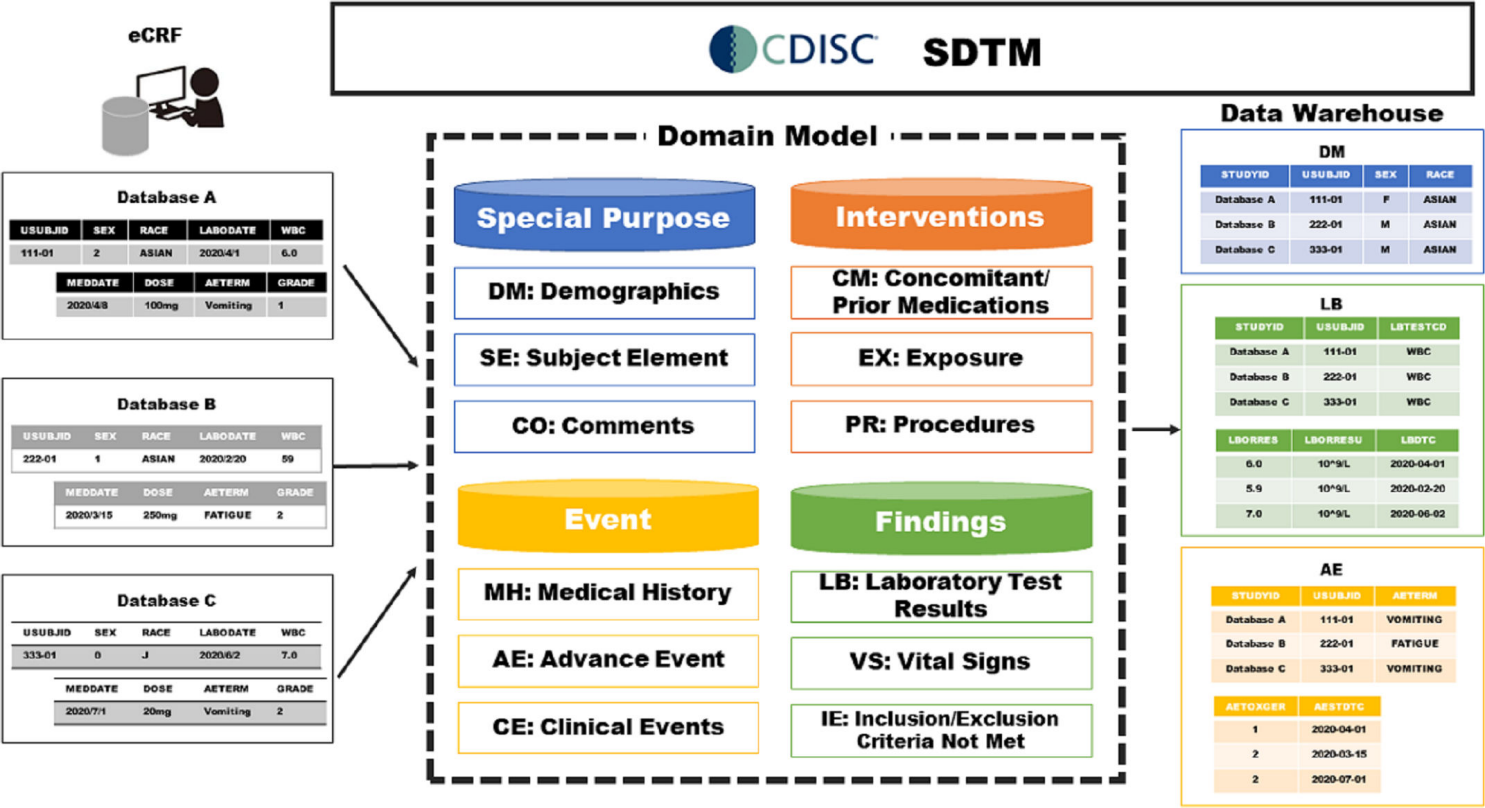
Clinical Data Interchange Standards Consortium (CDISC) Study Data Tabulation Model (SDTM) concepts. eCRF: electronic case report form.

The Clinical Data Interchange Standards Consortium (CDISC) is a nonprofit, global organization that has developed several data standards to streamline clinical research [4]. The Study Data Tabulation Model (SDTM) is a data standard model for the sharing and integration of research data, which was initially developed to standardize the tabulation of clinical trial data submitted to the Food and Drug Administration (FDA) [5]. The concept of the CDISC SDTM is shown in Figure 1. The CDISC SDTM consists of several domains derived from clinical aspects, and each domain is identified by a unique 2-letter code [6]. Metadata are described in the data definition document named “Define” that is submitted with the data to regulatory authorities [7]. Each data item collected in different databases, using the SDTM and Define.xml, enables one to unify variable names and codes easily. Clinical research data warehouses using the CDISC SDTM are considered useful for data sharing in academic research.

The integration of multiple data sets is difficult even among studies focused on the same disease. Major hurdles of data integration include the lack of standardization for the data format, variable names, and variable codes. Due to these problems, the manual conversion of data involves a large workload, which is likely to incur human error. Because the standardization of variable names and codes makes it easy to build a statistical data set, the CDISC SDTM provides a unique solution for database integration. However, many cohort studies have been conducted using a paper case report form (CRF) and formatted into data sets as a comma-separated values file or a spreadsheet file. It is difficult to convert these legacy data sets into the CDISC SDTM format because the variables need to refer to the CDISC variables and controlled terminology (CT).

Research Electronic Data Capture (REDCap) is an electronic data capture system developed by Vanderbilt University [8-10]. The “field annotation” function, introduced in REDCap version 6.5, can store meta-information for various standards related to clinical research, such as the CDISC, Systematized Nomenclature of Medicine (SNOMED), and Logical Observation Identifiers Names and Codes (LOINC). We previously developed “REDCap2SDTM,” a tool for parsing SDTM meta-information in the “field annotation” function and generating an XML file (Define-XML v2.0) with SDTM data [11,12]. This tool enables the efficient generation of SDTM data from multiple preexisting research data sets, and it has been validated for SDTM data generation based on hypothetical clinical trial data. However, only a few data integration projects using an actual research data set were carried out [13-15].

The purpose of this project was to design a practical working model for integrating preexisting databases using the CDISC SDTM. Here, we report the pragmatic conversion of multiple preexisting databases based on the CDISC SDTM format.

## Methods

### Ethical Considerations

Since this study was conducted on the structure of the database and not on patients, this study is outside the scope of ethical guidelines.

### Project Structure

**Figure 2.**
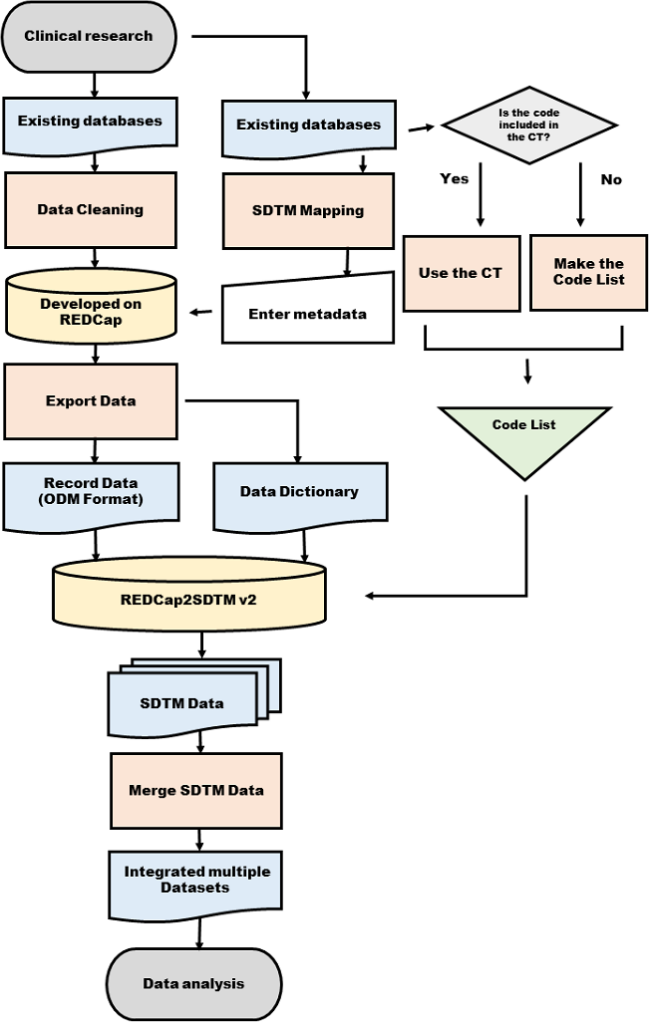
Flowchart of the data integration. CT: controlled terminology; ODM: Operational Data Model; REDCap: Research Electronic Data Capture; SDTM: Study Data Tabulation Model.

This project required multiple skill sets. A board-certified nephrologist (Japanese Society of Nephrology) with expertise in immunoglobulin A (IgA) nephropathy (including patient characteristics, laboratory data, and disease-specific items) confirmed the data structure in detail and constructed the independent database in REDCap. In parallel, a clinical data manager with CDISC SDTM expertise set the SDTM metadata in each variable. We outsourced the modification of REDCap2SDTM to a contract research organization to improve the efficiency of the SDTM data generation. The diagram of the study structure is shown in Figure 2.

### Data Source

IgA nephropathy is the most common type of chronic glomerulonephritis in Japan. IgA nephropathy is a refractory disease in which 30% to 40% of patients reach end-stage renal failure after approximately 20 years [16]. Various clinical features and a chronic course are the hallmark of this disease; therefore, a database that collects multiple items and a prognosis is needed. To date, the IgA Nephropathy Working Group in Progressive Renal Diseases Research, affiliated with Research on Intractable Diseases from the Ministry of Health, Labor and Welfare of Japan, has conducted 3 cohort studies with over 1000 participants in each cohort. However, the collected items and data structures in each cohort study were not standardized, making the construction of an integrated database difficult. The number of collected items and the data structure of each cohort are shown in Table 1.

**Table 1.** Characteristics of each cohort studies.

Cohort	Items in the data set, n	Sites, n	Data structure
A	57	6	Vertical format: with repeated measurement data
B	65	6	Horizontal format: no repeated measurement data
C	582	42	Horizontal format: no repeated measurement data

### Outline of Multiple Database Integration Work

The integration of multiple preexisting databases comprised the following three phases: (1) the confirmation of the variables in detail, (2) SDTM mapping, and (3) SDTM data generation and integration. The details of each phase are given below.

#### Phase 1: Confirmation of the Variables in Detail

In most cases, variable names differ by study, and the types of data also vary (date, digits, categorical variable, etc). Therefore, we set common values between each database in this phase.

Preexisting research data are stored in various formats between studies, including spreadsheets with a horizontal data (denormalized) structure. Since many SDTM domains are defined by a vertical data (normalized) structure, the data structure was transformed.

The main tasks of this phase were as follows:

- Standardize the variables in detail: code categorical data and nominal variables, unify date types (eg, YYYY/MM/DD), and improve the data format and the number of digits in clinical laboratory data in each data set
- Manage the data structure: transform repeated data from a horizontal structure to a vertical structure
- Validate the definition of variables: clarify data definitions and create a definition document in cooperation with specialists

#### Phase 2: SDTM Mapping

The CDISC has CT [17], and the terms used for each variable are specified in the *SDTM Implementation Guide* [6]. Through the use of CT, variables that were arbitrarily coded in different data sets can be derived as the same code. For example, if 1 data set coded male individuals as 1 and female individuals as 2 and another data set coded male individuals as 0 and female individuals as 1, the CT would code male individuals as “M” and female individuals as “F.” Therefore, the SDTM format data sets derived “M” for male individuals and “F” for female individuals. However, not all codes have specified CT, and coding lists for variables that are not specified must be created.

The domain model of the SDTM has a fixed domain of evaluation items to be stored. Therefore, each item in the data set must be mapped to the appropriate domain. For example, the “DM” domain contains the background of the patients (demographics), which includes age, sex, and race. The variable names were specified in each domain of the SDTM, for example, “SEX” for sex and “LBORRES” for laboratory results. Items with a unique code, such as sex, do not require a test code; the metadata are defined by the domain name “DM,” and the variable is named “SEX.” For items with various kinds of values, such as serum creatinine, a test code needs to be specified. For example, the meta-information of the creatinine test value must be defined by the domain name “LB,” the variable name “LBORRES,” and the test code “CREAT.” In addition, disease-specific end points are not defined in the standard domain of the SDTM. The SDTM does not allow new variables to be added arbitrarily; therefore, new variables must be defined in conjunction with the parent record using “supplemental qualifiers.” We determined the SDTM test code based on the appropriate code list from the SDTM CT.

Generally, in clinical studies, nominal scales (eg, male and female) are replaced by codes in the analysis. The method of assigning the code differs depending on the research and the data set, and recoding is necessary during database integration. We set both the domain and the meta-information of each data set based on the definitions confirmed in phase 1.

The main tasks of this phase were as follows:

- Recoding: map nominal variables and codes according to the CT or custom coding lists
- SDTM metadata mapping: map existing data variables to the SDTM domains

#### Phase 3: Generate SDTM Data in the Operational Data Model Format

**Figure 3.**
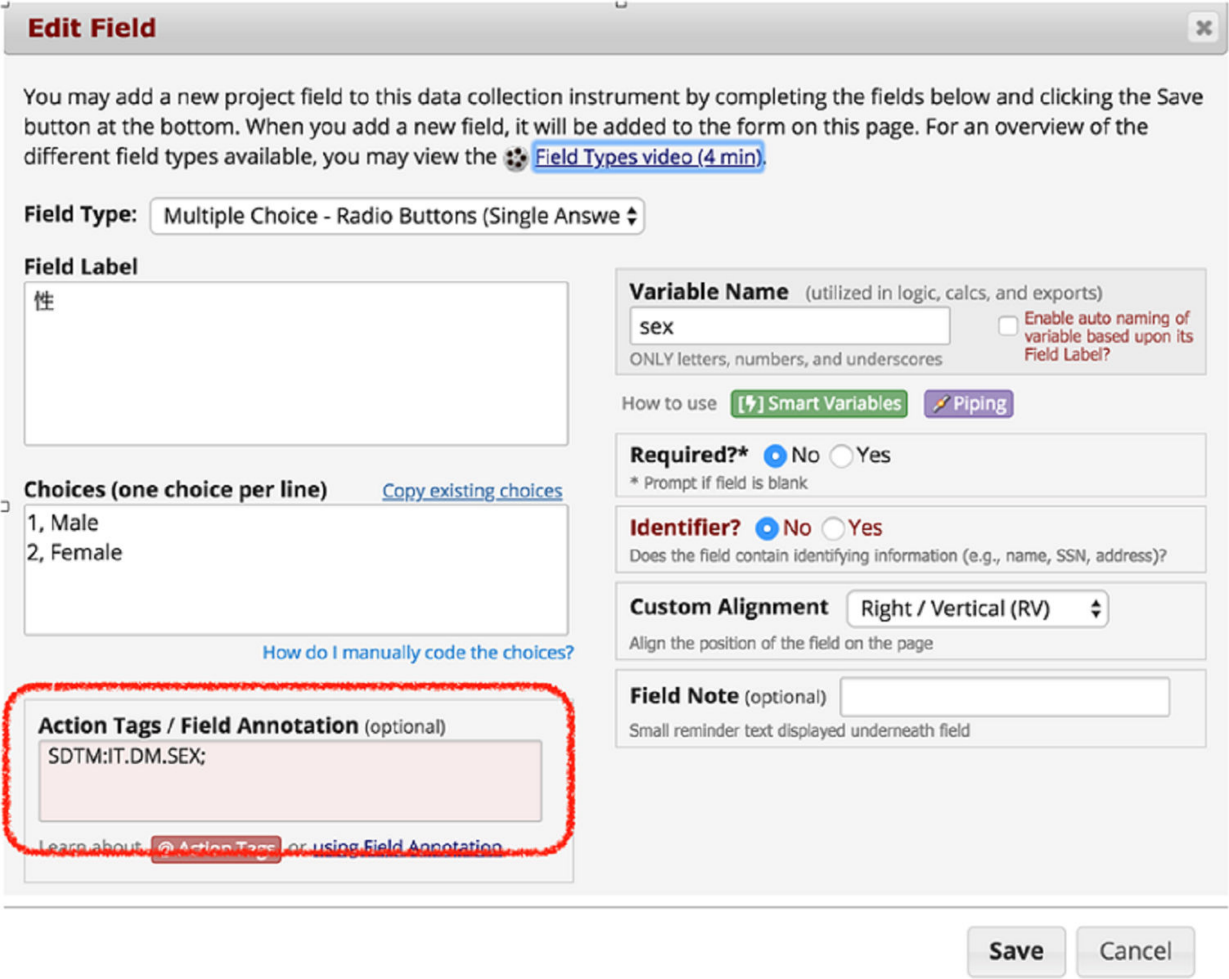
Screenshot of “field annotation.” SDTM: Study Data Tabulation Model.

In this phase, the SDTM metadata were manually set in the “field annotation” function (Figure 3). Subsequently, the data, including the data dictionary with the SDTM metadata, were downloaded in the Operational Data Model (ODM) format with SDTM metadata. Finally, REDCap2SDTM automatically generated each data set in the ODM format with the SDTM metadata.

Common items in all data sets must be assigned the same metadata. Therefore, it is necessary to identify common items in all data sets to confirm the consistency of the metadata.

The main tasks of this phase were listed as follows:

- Check the consistency of the metadata: unify common items between each data set
- Generate SDTM metadata from each database: download and record data and data dictionaries and generate SDTM metadata with REDCap2SDTM
- Output SDTM metadata in the ODM format: retrieve the SDTM metadata output from REDCap2SDTM

**Figure 4.**
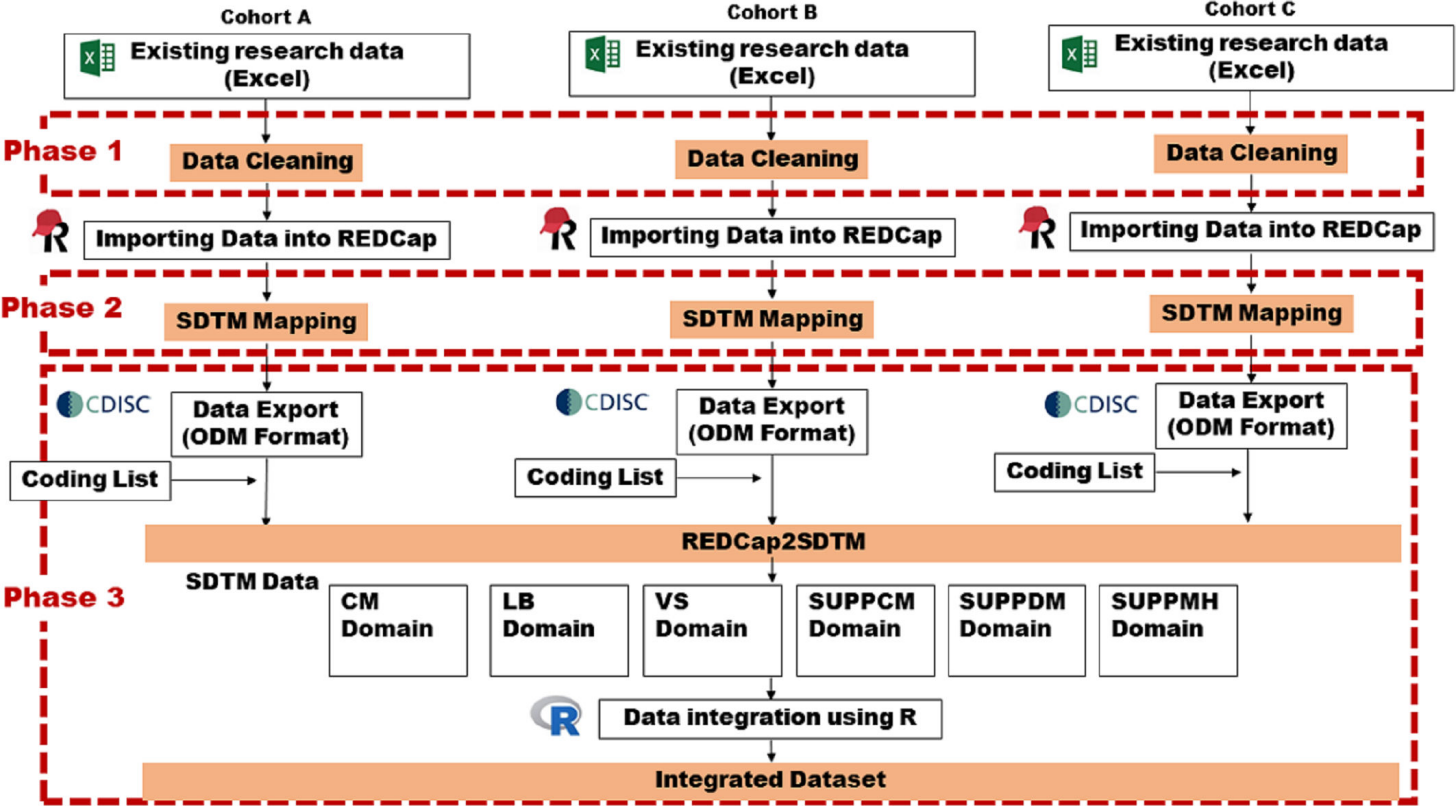
Diagram of the study structure. CDISC: Clinical Data Interchange Standards Consortium; ODM: Operational Data Model; REDCap: Research Electronic Data Capture; SDTM: Study Data Tabulation Model.

A summary of the data integration process is shown in Figure 4.

### REDCap2SDTM Version 2

REDCap2SDTM combined formatted ODM data that were embedded with 3 pieces of metadata (ie, SDTM domain name, variable name, and test code) into the field annotation of REDCap as the metadata corresponding to the variable name of the data set, to convert the database into the SDTM format. This tool dynamically generates SDTM data and a Define.xml file by parsing. The syntax of the meta-information is the CDISC Define-XML version 2.0 “ItemDef element.” REDCap2SDTM version 2 parses the object identifier attribute value and uses that information for mapping (eg, “IT.VS.VSORRES. SYSBP” and “IT.AE. AETERM”) [11,12].

The CDISC ODM is a vendor-neutral, platform-independent data format for exchanging and storing clinical research data and metadata that can be shared between different software systems [18]. In this case, we modified REDCap2SDTM to adopt the CDISC ODM format (REDCap2SDTM version 2; Multimedia Appendix 1). Due to this modification, REDCap2SDTM version 2 could convert the SDTM data to the ODM format and could expand to handle variables across multiple domains.

## Results

This project was conducted from July 2018 to January 2019. Items were selected for integration in the SDTM metadata based on the opinions of the board-certified nephrologist.

Regarding disease-specific items, the histological classification of the disease was defined in the “SUPPMH” domain; items related to family history were defined in the “SUPPDM” domain, and the number of steroid pulse therapies was defined in the “SUPPCM” domain. The following domains were generated in this study: “DM” (demographics), “CM” (concomitant medications), “LB” (laboratory test results), “VS” (vital signs), “SUPPCM,” “SUPPDM,” and “SUPPMH.”

The preexisting database included 57 total items for cohort A, 65 total items for cohort B, and 582 total items for cohort C. The metadata were set for 40 items for cohort A, 18 items for cohort B, and 102 items for cohort C. We found 17 common items. Finally, a total of 119 items were set for the SDTM metadata. Of these, 56 items used the nominal scale, 48 items could be recoded using CT, and 8 items required independently created code lists. Disease-specific items, such as the pathological classification based on the clinical guidelines for IgA nephropathy in Japan [19] and the Oxford classification [20], required their own code list. Table 2 lists the SDTM metadata of key items.

The data dictionary and ODM data were outputted from REDCap, and REDCap2SDTM version 2 was used to output the data in the SDTM format. The items defined by individual names in each database were collated based on the metadata by the CDISC SDTM.

**Table 2.** SDTM^a^ metadata of the key items.

Items		Cohort A	Cohort B	Cohort C	SDTM metadata
Sex		Sex	Sex	Sex	DM.SEX
Birthday		Birth_date(Y/M/D)	Birth_date(Y/M/D)	birth date	DM.BRTHDTC
Age		—^b^	—	Age	DM.AGE
**Vital sign**					
	Systolic blood pressure	sbp_bx	sbp_bx	Sbp	VS.VSORRES.SYSBP
**Concomitant drugs**					
	Renin-angiotensin system inhibitor	rasb_prior	rasb_prior	Ras	CM.CMOCCUR.RAS
	Date of first immunosuppressants	—	—	Day	CM.CMSTDTC.PSL
	Prednisolone (yes or no)	IS_bx	fuSteroids_bx	ral steroid p or a	CM.CMOCCUR.PSL
	Immunosuppressants without prednisolone (yes or no)	Non_steroid_IS	—	immuno therapy	CM.CMOCCUR.PSLOTH
**Tonsillectomy**					
	Tonsillectomy (yes or no)	tonsillectomy	fu_tonsillectomy	Tonsil	SUPPMH.QNAM.OPE
	Date of tonsillectomy	tonsillectomy_dt	—	tonsil date	SUPPMH.QNAM.OPEDATE
**Laboratory examinations**					
	Date of kidney biopsy	date_bx	date_bx	kidney_biopsy_date	LB.LBDTC.BIOPSY
	Serum creatinine	Creatinine	—	Cr	LB.LBORRES.CREAT
	eGFR^c^	eGFR	gfr_bx_provided	Egfr	LB.LBORRES.EGFR
	Urinary protein (spot)	uprot_bx	uprot	urinprotein1	LB.LBORRES.PROT1
	Urinary protein (24 h)	uprot_24h_bx_provided	uprot_24h	Urinprotein	LB.LBORRES.PROT24
**Pathological findings**					
	Oxford classification: mesangial hypercellularity (M)	m	m	Oxford1	SUPPMH.QNAM.M
	Oxford classification: endocapillary hypercellularity (E)	e	e	Oxford2	SUPPMH.QNAM.E
	Oxford classification: segmental glomerulosclerosis (S)	s	s	Oxford3	SUPPMH.QNAM.S
	Oxford classification: tubular atrophy/interstitial fibrosis (T)	t	t	Oxford4	SUPPMH.QNAM.T

a SDTM: Study Data Tabulation Model.

b Not available.

c eGFR: estimated glomerular filtration rate.

## Discussion

### Strength of This Study

Integrating multiple preexisting databases through collaboration between the disease specialist and clinical data manager enabled the use of legacy data. Our project suggested that properly defining CDISC SDTM metadata allowed for the integration of multiple preexisting databases. In this paper, we focused on the technical aspect. Although the utility of this concept has been verified with hypothetical data, there are few reports that generate SDTM data from actual clinical databases focused on technical aspects in detail.

### The CDISC SDTM

The definition of metadata using the CDISC SDTM is important. The CDISC is a nonprofit, global organization that consists of pharmaceutical companies, contract research organizations, academic research organizations, and IT vendors. Pharmaceutical companies and contract research organizations account for 34% of the entities within the CDISC, whereas academic research organizations account for only 7% [21]. This imbalance may have arisen because those submitting a regulatory application to the FDA or the Pharmaceuticals and Medical Device Agency are required to comply with CDISC standards [22]. Therefore, there is a strong awareness of the CDISC as a tool for regulatory submissions, but few researchers are aware that the CDISC SDTM concept can be used to standardize data.

The mission of the CDISC is to develop and support global, platform-independent data standards that enable information system interoperability to improve medical research and related areas of health care. Following this statement, we have succeeded in integrating 3 databases by incorporating the CDISC SDTM concept into the standardization of multiple databases. Since this database complies with the standardization of the CDISC SDTM, this integrated database can be compared to other clinical trials or it can be used as a historical control. Our study shows that the CDISC SDTM is not only a necessary tool for applying for the approval of regulatory submissions but also for data standardization and integration. In recent years, the CDISC has partnered with REDCap to make Clinical Data Acquisition Standards Harmonization eCRF metadata available in the REDCap Shared Instrument Library [22,23]. It is expected that the researchers will be able to import CDISC SDTM metadata directly into their REDCap projects for immediate use in clinical trial data collection. In the future, CDISC SDTM data will be generated more easily.

We were able to develop the methodology for integrating multiple preexisting databases in just 6 months. This timely integration was due to the collaboration of specialists in the disease area and a data manager familiar with the CDISC SDTM, allowing each phase to proceed simultaneously and resulting in a fast integration time. Inconsistencies in the coding method of the nominal scale hindered the integration of multiple databases. However, in this study, codes defined individually for each database were automatically recoded to substantially reduce the required work hours, which also contributed to the fast integration time. When coding terms not defined by CT, such as terms that are specific to the disease area, a code list should be created following thorough discussions with specialists, referring to therapeutic area standards [24]. It is important to improve work efficiency by making the best use of existing materials. Although the preexisting databases were integrated in this study, even in cases where the data were updated longitudinally, it is possible to integrate data with the SDTM, provided that the meta-information for the evaluated item is defined.

Currently, there are several medical standards. Observational Medical Outcomes Partnership (OMOP), which is managed by Observational Health Data Science and Informatics [25], aimed to standardize interoperability observational databases such as electric medical records and claim data. HL7 Fast Healthcare Interoperability Resources (FHIR) [26] is the standard for medical information exchange. In this study, we used the CDISC SDTM because, at the time, it was the most widely used standard with many accumulated findings. We plan to expand this project to support the OMOP Common Data Model and FHIR in the future.

### Issues for Integration

We observed the following points when integrating the preexisting clinical databases: (1) the variability of the collected items and (2) the complexity of the test code. The items in the preexisting cohort studies used in this project were not standardized and were not defined in detail; therefore, we clarified the meaning of the variables based on expert opinions. Clarifying data definitions is difficult for data managers who lack the requisite background knowledge.

In addition, we were faced with large differences in the number of items collected from each preexisting cohort study. As previously mentioned, 57 and 65 items were collected in cohorts A and B, respectively, far fewer than the 582 items collected in cohort C, which included data related to concomitant medications. However, because information on concomitant medications is often missing, it is considered a difficult item to use for analysis. Generally, information on concomitant medications is not used for analysis and is not collected in precise clinical trials. To avoid complications in the integration process, information collected on concomitant medications should focus on those related to the disease area or should be divided into categories prior to collection. These findings were obtained by scrutinizing the differences in the items collected in each database prior to generating the metadata.

The complexity of the test code was clarified during the generation of the metadata. As described above, the amount of the urinary protein was defined as both “PROT” and “PROT24.” Because the details of proteinuria are not defined in CT, there is a risk for inappropriate metadata. These findings suggest that the generation of metadata requires a deep understanding of the disease in addition to the concepts of the CDISC SDTM. In this study, the clinical data manager who had knowledge of the CDISC SDTM was responsible for generating the metadata in collaboration with a specialist in the disease area. Currently, clinical data managers primarily play an active role in prospective clinical trials. Thus, the main responsibilities of the clinical data manager are planning the clinical trial, assisting with the creation of the protocol and CRF, cleaning the data, confirming data consistency, and managing data quality in clinical trials. We believe that the clinical data manager will play an important role for data integration projects in the near future. Collaborations between the clinical data manager and the disease specialist will likely become even more important.

### Limitations

This project had several limitations. First, the data of the cohort studies did not cover all domains of this disease. In the future, we would like to increase the number of integration examples and generalize the program to cover all domains. Second, a great deal of time was spent manually setting the metadata. In the future, it may be beneficial to automatically refer to the shared metadata from the CDISC Library or to develop a tool that allows artificial intelligence to suggest the metadata using therapeutic area standards. Third, REDCap2SDTM version 2 required input for the ODM format. Several programs that generate ODM or Define-XML data from a spreadsheet are available from the CDISC Open Source Alliance [27]. We will consider embedding these programs into REDCap2SDTM version 2 in the future.

### Conclusion

Our results suggest that the CDISC SDTM is useful for integrating multiple preexisting databases with variable names and codes. We hope that this research will contribute to the use of legacy data sets.

## Supplementary material

10.2196/46725 Multimedia Appendix 1 The package of REDCap2SDTM version 2.

## Abbreviations

CDISC: Clinical Data Interchange Standards Consortium
CRF: case report form
CT: controlled terminology
FDA: Food and Drug Administration
FHIR: Fast Healthcare Interoperability Resources
ICMJE: International Committee of Medical Journal Editors
IgA: immunoglobulin A
LOINC: Logical Observation Identifiers Names and Codes
ODM: Operational Data Model
OMOP: Observational Medical Outcomes Partnership
REDCap: Research Electronic Data Capture
SDTM: Study Data Tabulation Model
SNOMED: Systematized Nomenclature of Medicine
